# Supplementary material for: Lactobacillus iners dominates the vaginal microbiota of healthy Italian women of reproductive age
Source: mSystems. 2025 Nov 25;10(12):e00983-25. doi: 10.1128/msystems.00983-25 (PMC12710342; doi:10.1128/msystems.00983-25)

**Figure S1: Taxonomic Comparison Between GTDB and SILVA Databases used for the Reclassification of *Bifidobacterium vaginale* as *Gardnerella vaginalis.***

In **(A)** we report the number of ASVs and corresponding species identified within the genus *Bifidobacterium* using the GTDB database, and their respective classifications for the same ASVs using the SILVA database. Each bar represents the number of ASVs assigned to each taxon. In **(B)** we show the correspondence between taxonomic classifications obtained with GTDB and SILVA. Each cell shows the number of ASVs shared between the two databases.


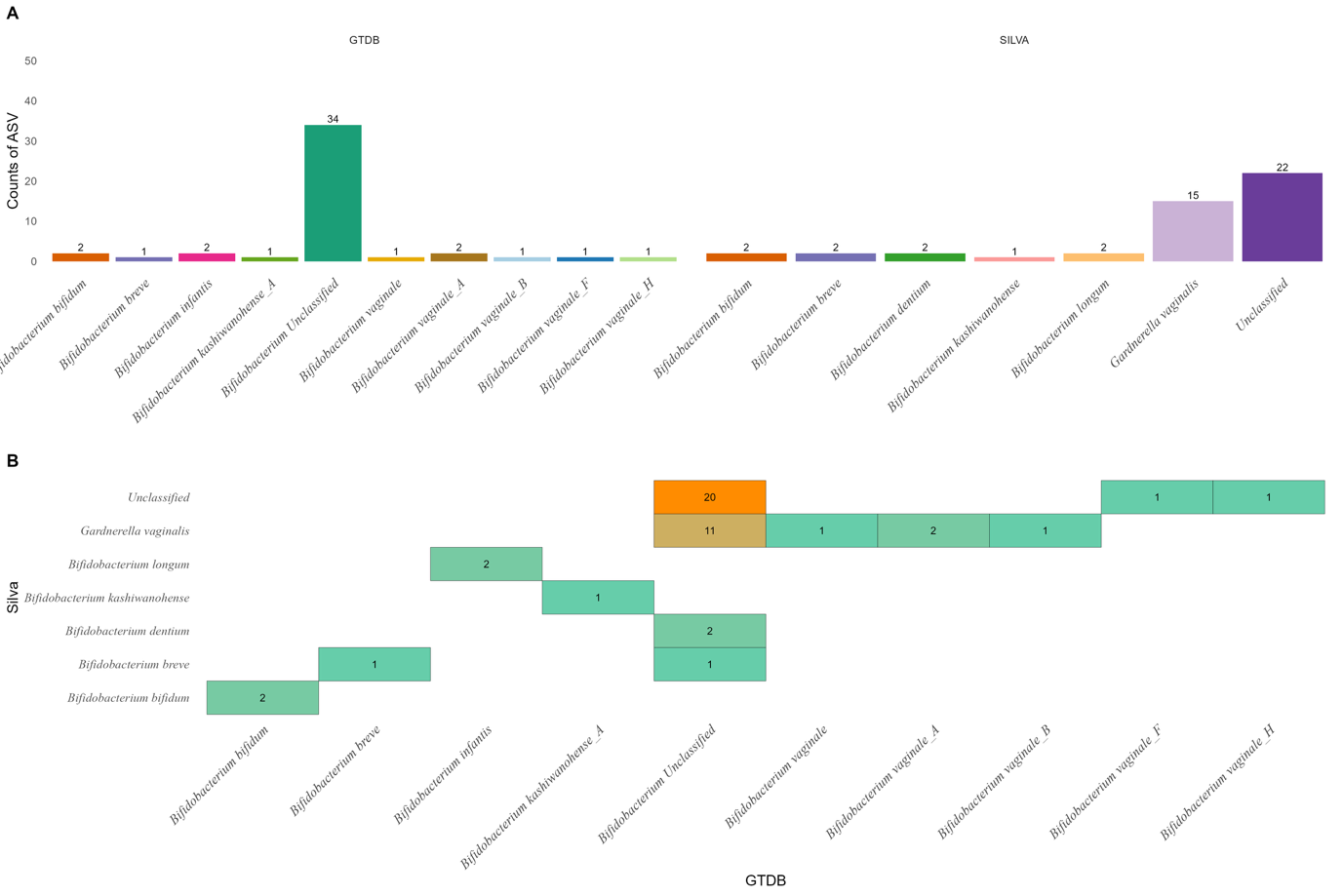


**Figure S2: t-SNE representation of vaginal microbiome composition**

This figure shows t-SNE representation of the vaginal microbiome profiles, where each point corresponds to a sample. In each panel, a different colour coding is used to visualize different information. In **(A)** points are coloured by the most abundant taxon – at the species level for *Lactobacillus*, and at the genus level for other bacteria; in **(B)** points are coloured according to the second most abundant taxon – at the species level for Lactobacillus, and at the genus level for other bacteria ; in **(C)** points are coloured according to the relative abundance of the most dominant species is in that specific sample; in **(D)** points are coloured according to the sub-CST classification.


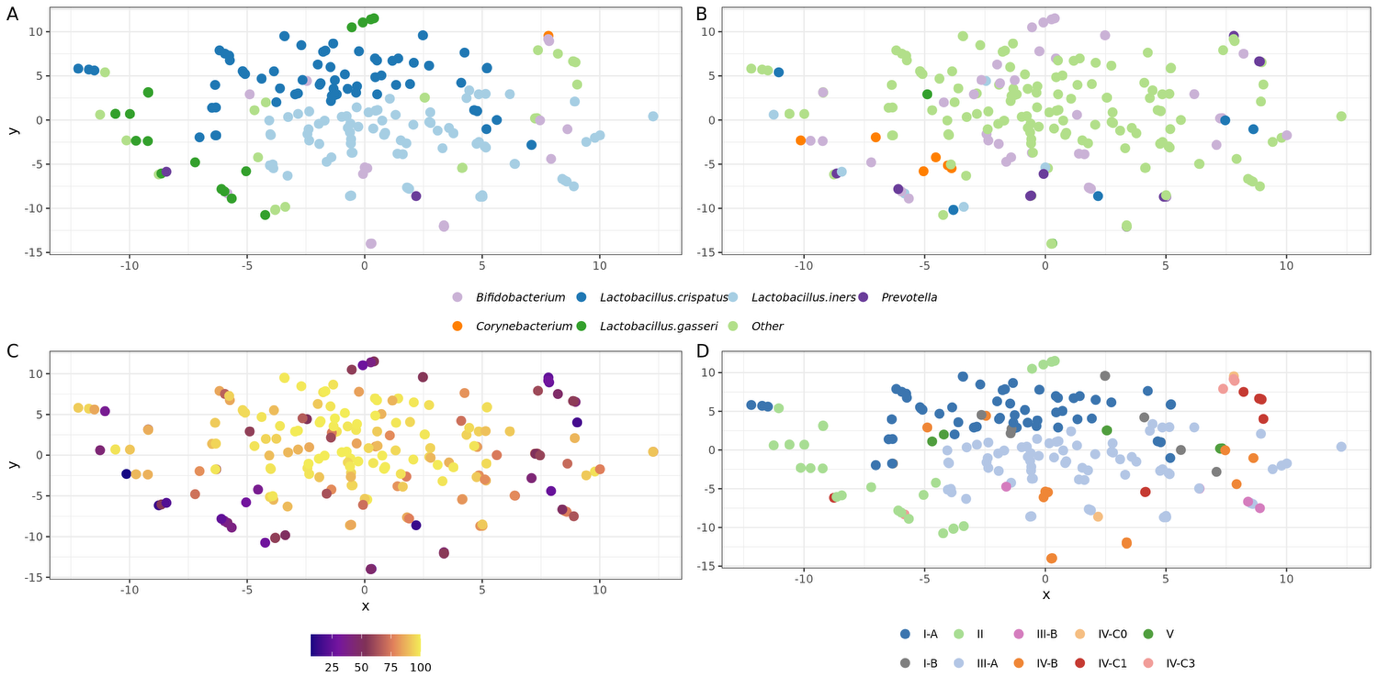


**Figure S3. Most abundant bacterial species.**

Bar plot showing the relative abundance (in percentage) of the 10 most abundant bacterial species. The y-axis shows the relative abundance (in percentage) with samples analysed in the x-axis. Samples are ordered according to volunteers’ ID and visit number. The coloured bar shows samples with QC and vaginal microbiome profile (n=212) while the grey bars are samples for which we have no information (samples that either failed QC or for which no vaginal swab was available, n=32).


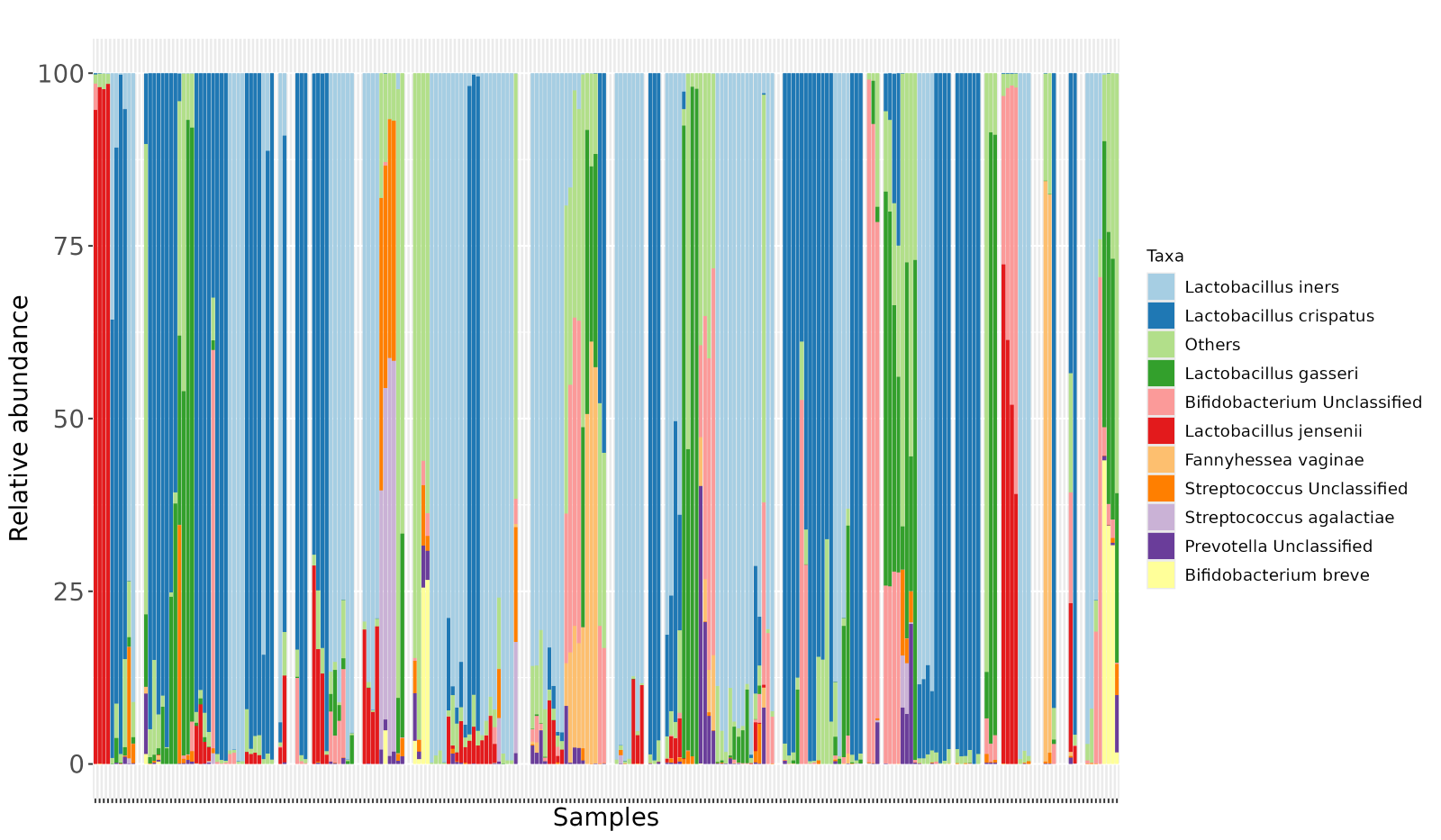


**Figure S4: Alpha diversity between menstrual cycle phases**

In **(A)** we show the distribution of Shannon index (H), which accounts for both species richness (number of taxa) and evenness (how evenly the counts are distributed among the taxa); low values (around zero) indicate low diversity, while higher values indicate greater diversity. In **(B)** we show the distribution of the Simpson index (D) which shows the dominance of species. Values close to 1 indicate low diversity (one species dominates) while values close to 0 indicate high diversity (no dominant species). The violins are shown for the four phases: Follicular (F), Ovulatory (O), Early Luteal (EL), and Late Luteal (LL) phases.


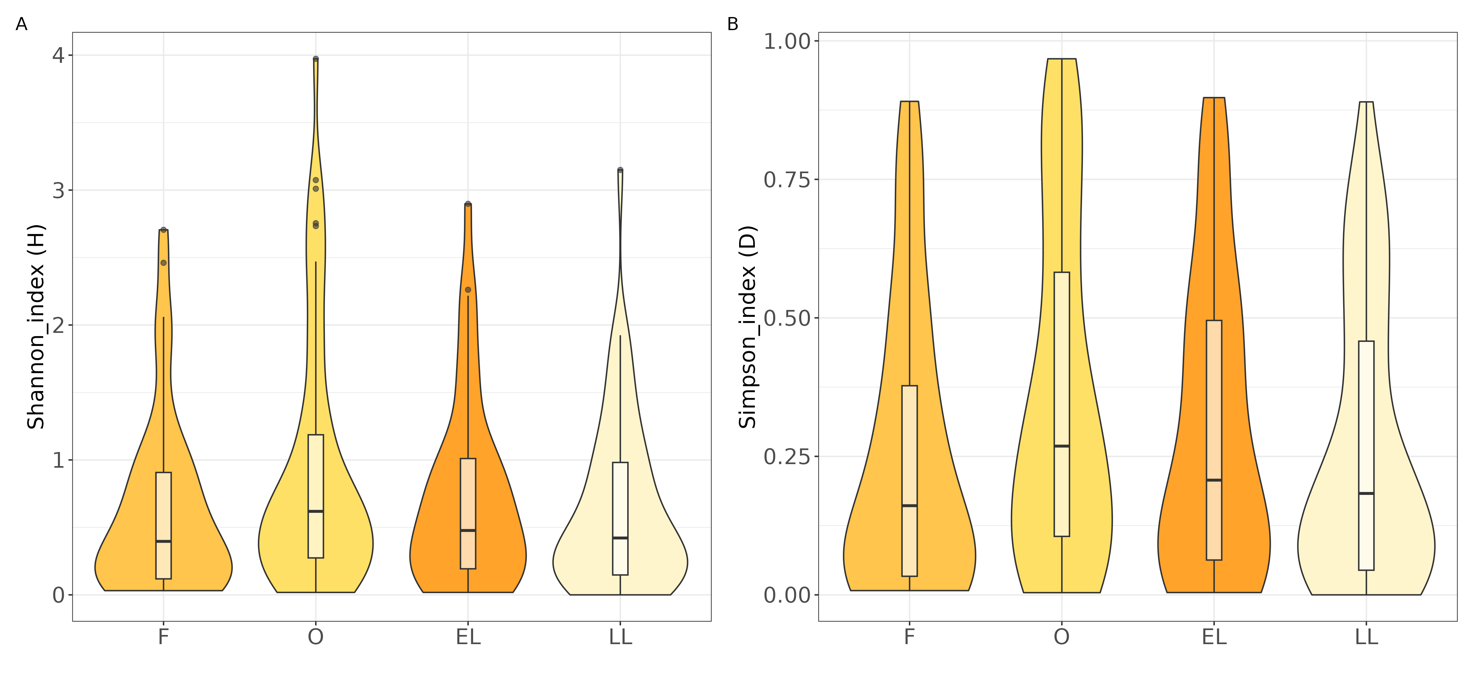


**Figure S5: Median beta diversity across menstrual cycle phases**

Median of Beta diversity across four phases: Follicular (F), Ovulatory (O), Early Luteal (EL), and Late Luteal (LL) phases. The black lines between violins indicate significant differences (*: 0.01 < p < 0.05). Pvalues were obtained using paired T-test.


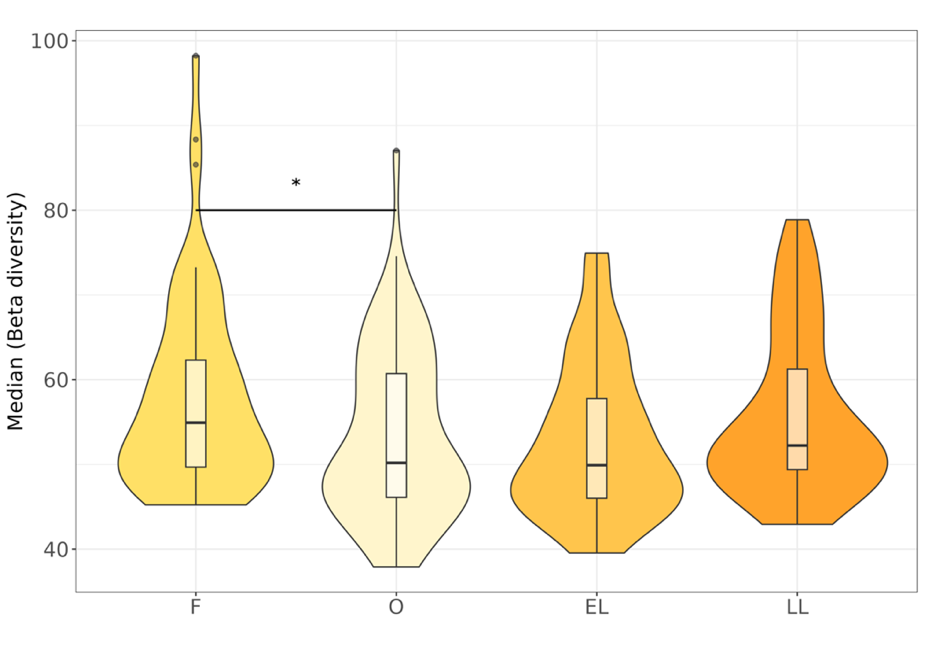


**Figure S6: Beta diversity distribution across menstrual cycle phases**

Beta diversity distribution across the four phases: Follicular (F), Ovulatory (O), Early Luteal (EL) and Late Luteal (LL) phases. The y-axis shows Euclidean distance. All pairwise comparisons were statistically significant (p < 0.05 with a Wilcoxon test and unpaired T-test).


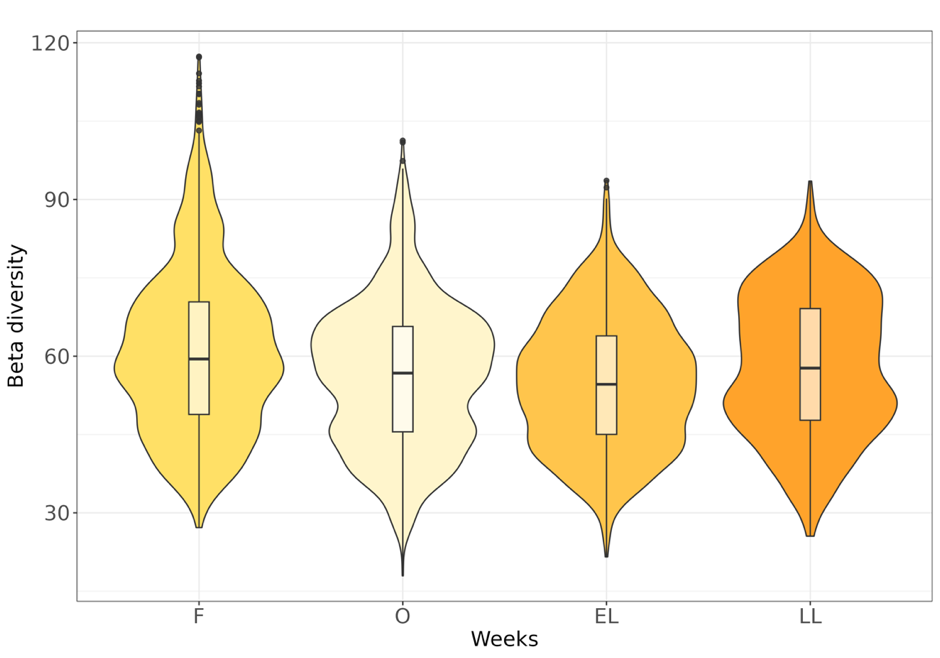


**Figure S7: Micobiome composition**

The stacked bar plot shows the relative abundance (in percentage) of the 10 most abundant fungi species. The y-axis shows the relative abundance (percentage) with the samples analysed in the x-axis. Bars are coloured only for samples for which more than 200 reads were detected ITS region (n=25), while the bars in grey depict samples without ITS-related fastQ or with less than 200 reads. Samples are ordered according to **Figure 3**.


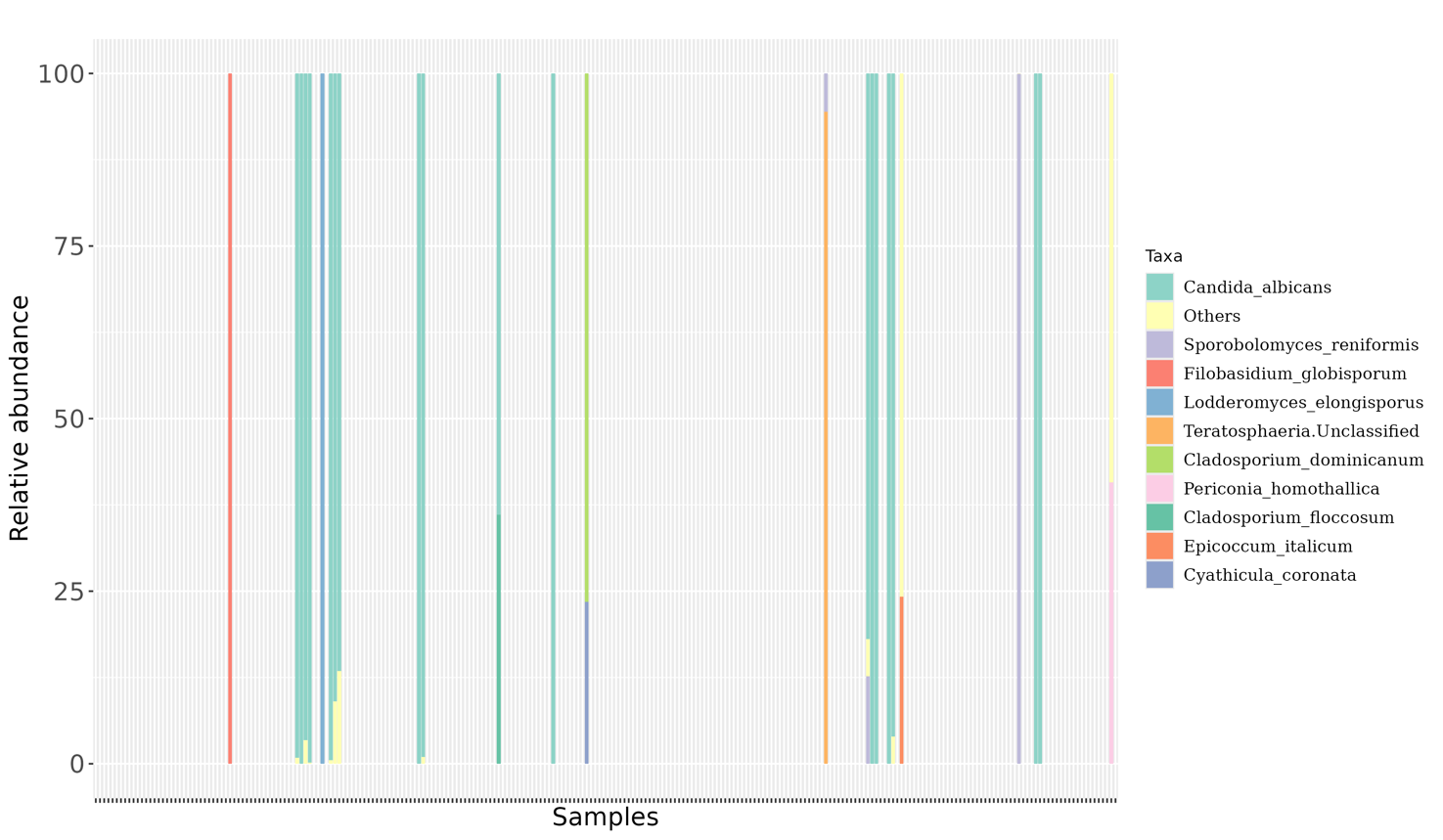


**Figure S8: Results of independence test**

Results of independence tests on Principal Coordinates Analysis (PCoA) at the genus and species classification levels. Each panel corresponds to data collected at a specific phase. The x-axis within each panel represents the specific PCoA axes. The colour scale, as detailed in the legend, indicates the significance levels of the p-values for the independence tests. Refer to Supplementary Table S5 for detailed descriptions of the categorical features involved.


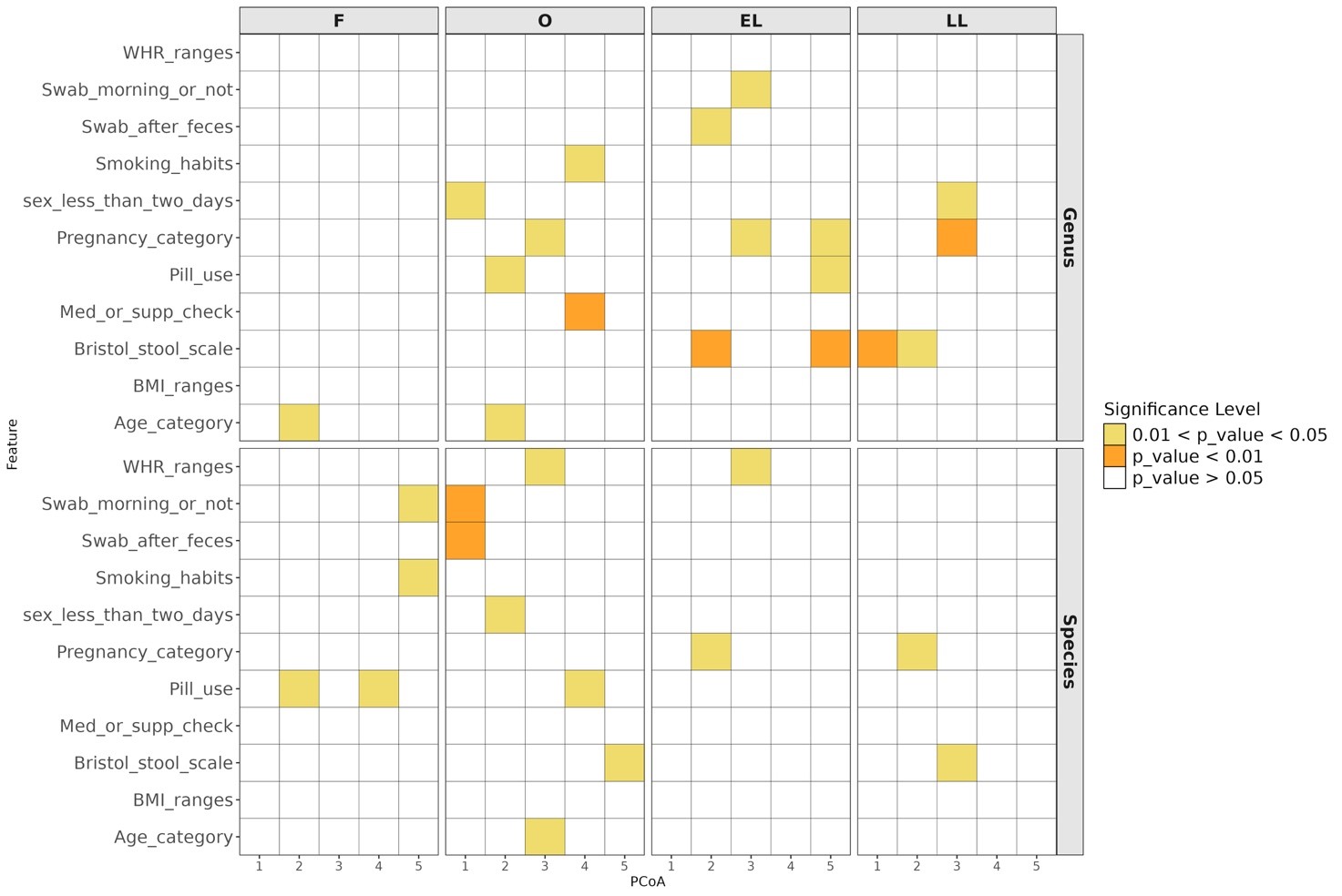


**Figure S9: Impact of having had children**

This figure shows the same PCoA space in both panels but coloured according to different type of information. We focused on PCoA axes that were significant in the PERMANOVA test. In **(A)** we show clusters of samples coloured and named according to the most of bacterial communities, with confidence ellipses representing the distribution of each group. In **(B)** we show the same PCoA plot coloured by pregnancy category (having had children in the past), with ellipses representing the dispersion of each group. Counts of each entry are shown in the upper left corner. The p-value of the Adonis test is shown in the plot.


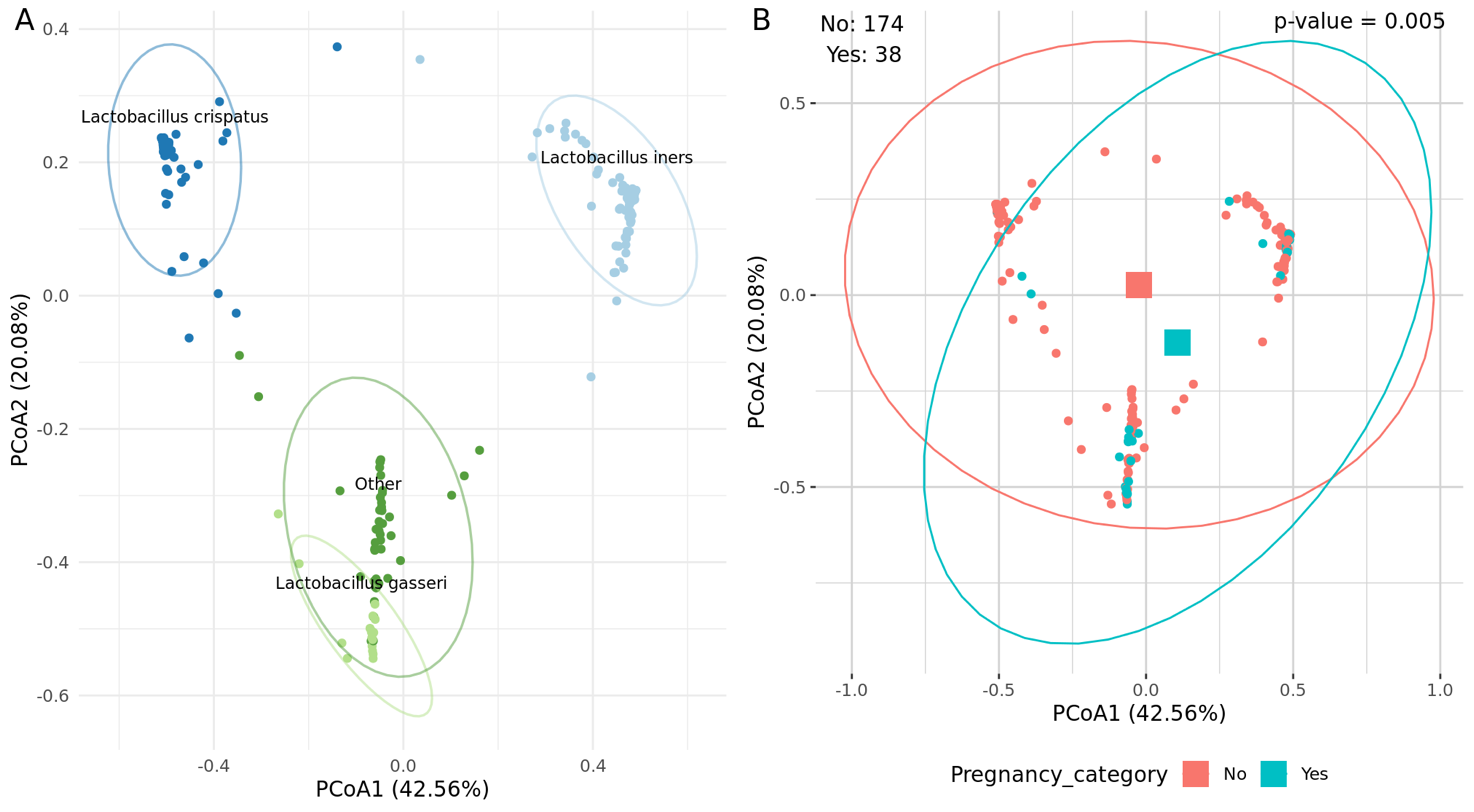


**Figure S10: Impact of different age categories**

This figure shows the same PCoA space in both panels but coloured according to different type of information. We focused on PCoA axes that were significant in the PERMANOVA test. In **(A)** we show clusters of samples coloured and named according to the most of bacterial communities, with confidence ellipses representing the distribution of each group. In **(B)** we show the same PCoA plot coloured by age categories, with ellipses representing the dispersion of each group. Counts of each entry are shown in the upper left corner, while the p-value of the Adonis test is shown in the plot.


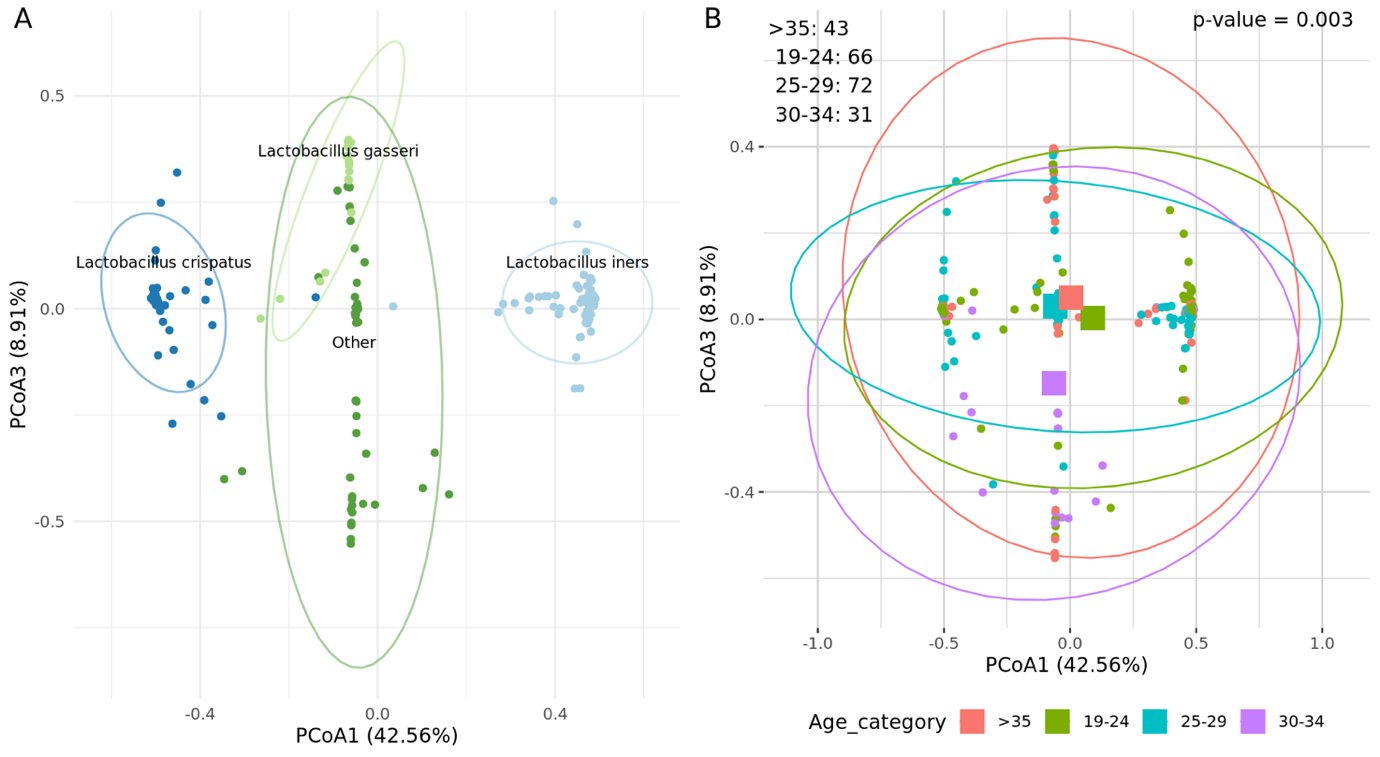


**Figure S11: Effect of collecting swabs after collection of feces**

This figure shows the same PCoA space in both panels but coloured according to different type of information. We focused on PCoA axes that were significant in the PERMANOVA test. In **(A)** we show clusters of samples coloured and named according to the most of bacterial communities, with confidence ellipses representing the distribution of each group. In **(B)** we show the same PCoA plot coloured by swab collection status after defecation (No, Yes, NA), with ellipses representing the dispersion of each group. Counts of each entry are shown in the upper left corner. The p-value of the Adonis test is shown in the upper right corner.


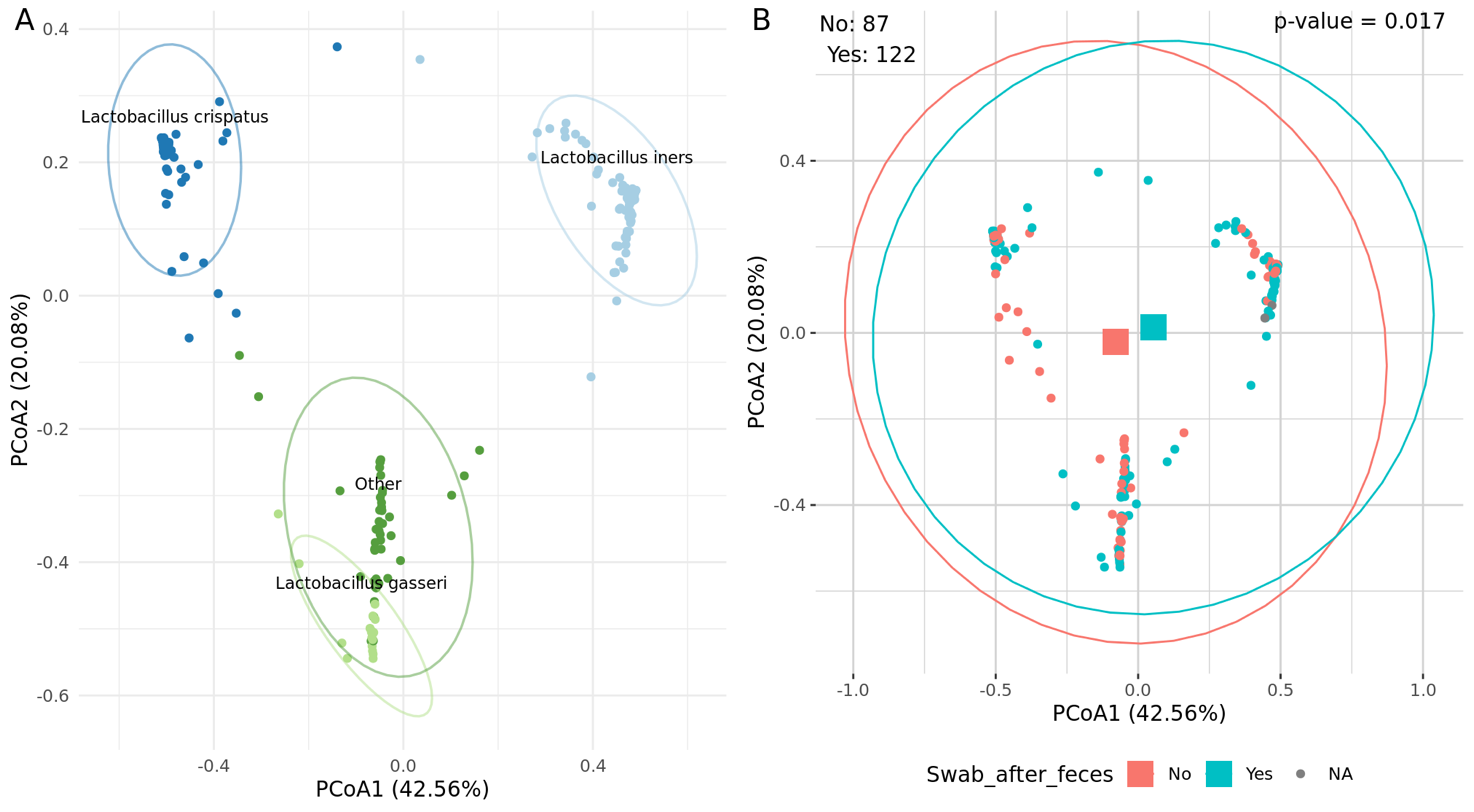


**Figure S12: Impact of time of vaginal swabs collection**

This figure shows the same PCoA space in both panels coloured according to different type of information. We focused on PCoA axes that were significant in the PERMANOVA test. In **(A)** we show clusters of samples coloured and named according to the most common bacterial communities, with confidence ellipses representing the distribution of each group. In **(B)** we show the same PCoA plot coloured this time by swab collection time (morning, not morning, NA), with ellipses representing the dispersion of each group. Counts of each entry are shown in the upper left corner. The p-value of the Adonis test is shown in the plot.


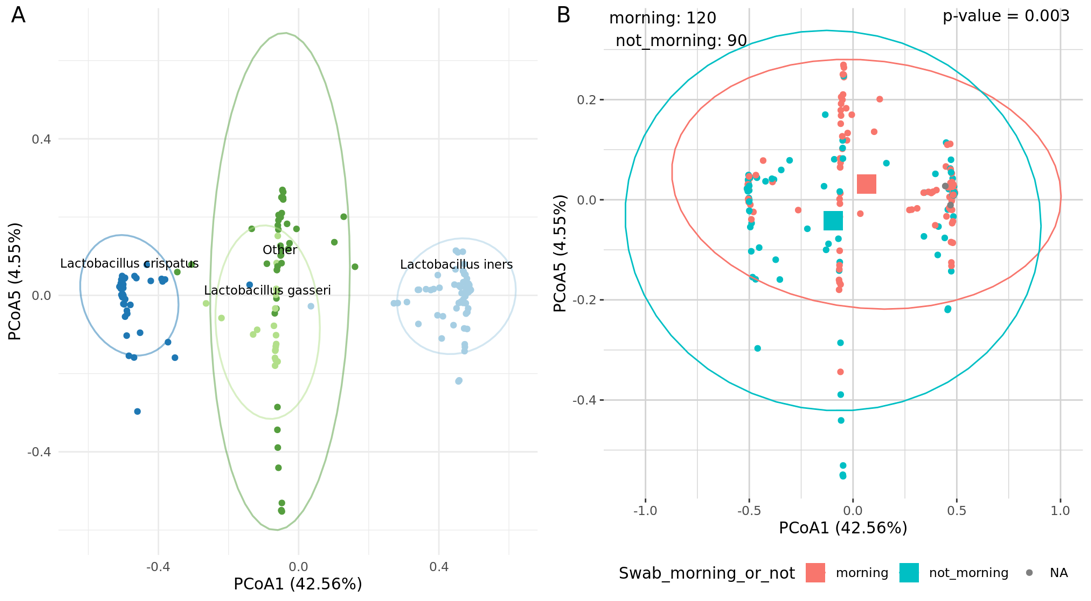


**Figure S13: Comparison of microbiomes profiled using 16S rRNA amplicon sequencing and WGS**

In **(A)** relative abundance of top 10 taxa detected in ten samples characterized using Whole-Genome Sequencing (WGS). In **(B)** 16S rRNA amplicon sequencing method.


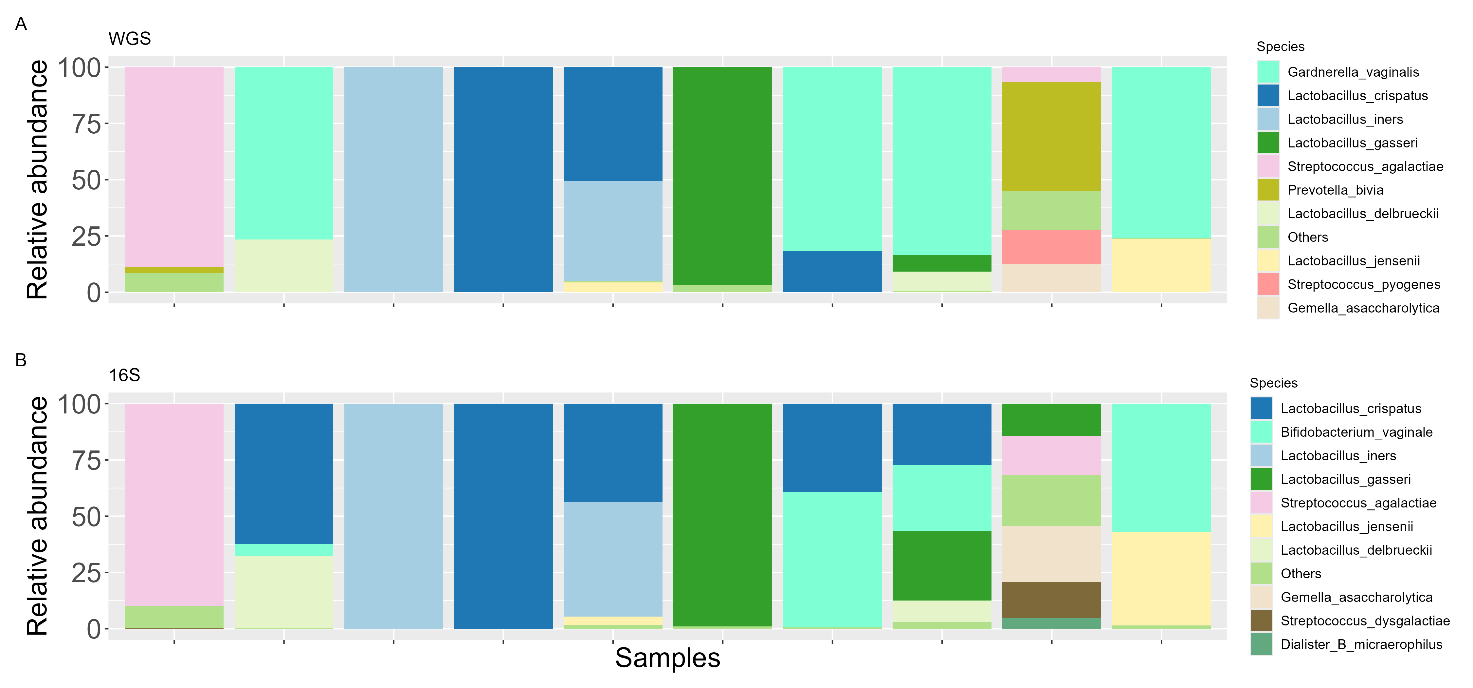


**Figure S14: Comparison between microbiome profiles obtained in the main and the subsampling-based analyses**

These scatterplots visualize the comparison between average relative abundance and prevalence of taxa obtained in the main analysis and those obtained when analysing the subsampled data set at 30k reads in **(A, C)** and 60k reads in **(B, D)**.


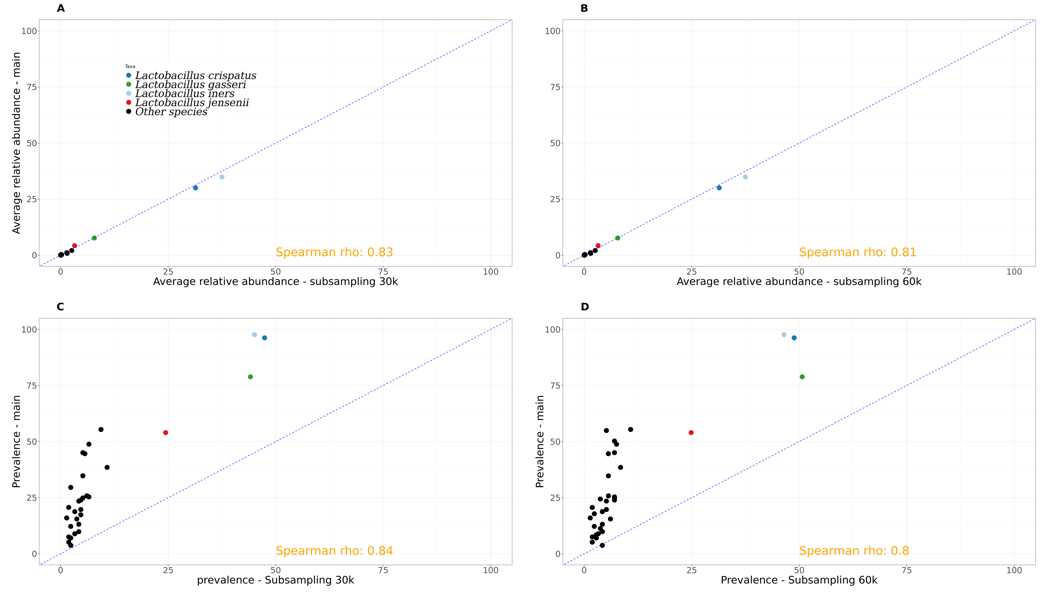


**Figure S15: Distribution of the CST (Community State Type)**
In **(A)** pie chart showing the percentages of each CST among all samples. In **(B)** percentage of each CST within each menstrual cycle phase.


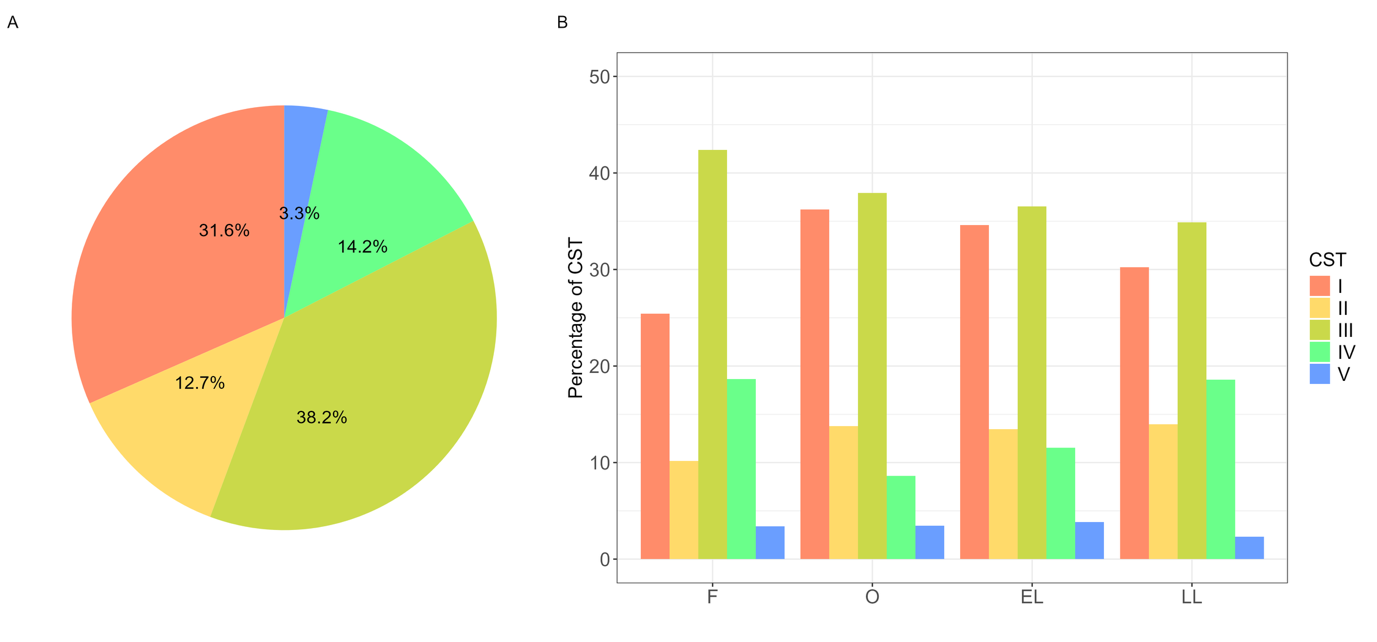


**Figure S16: Alluvial plot of CST.**

Alluvial plot visualising changes in CST (Community State Type) between menstrual cycle phases. Each column is a phase: Follicular (F), Ovulatory (O), Early Luteal (EL) and Late Luteal (LL) Phases. Color-coding indicates: changes occurred in at least one phase (CH), complete data available (9/61); CH + NA: changes occurred in at least one phase, missing samples in some phases (2/61); NO CH: No changes occurred, complete data available (32/61); NO CH + NA: No changes occurred, but missing samples in some phases (18/61).


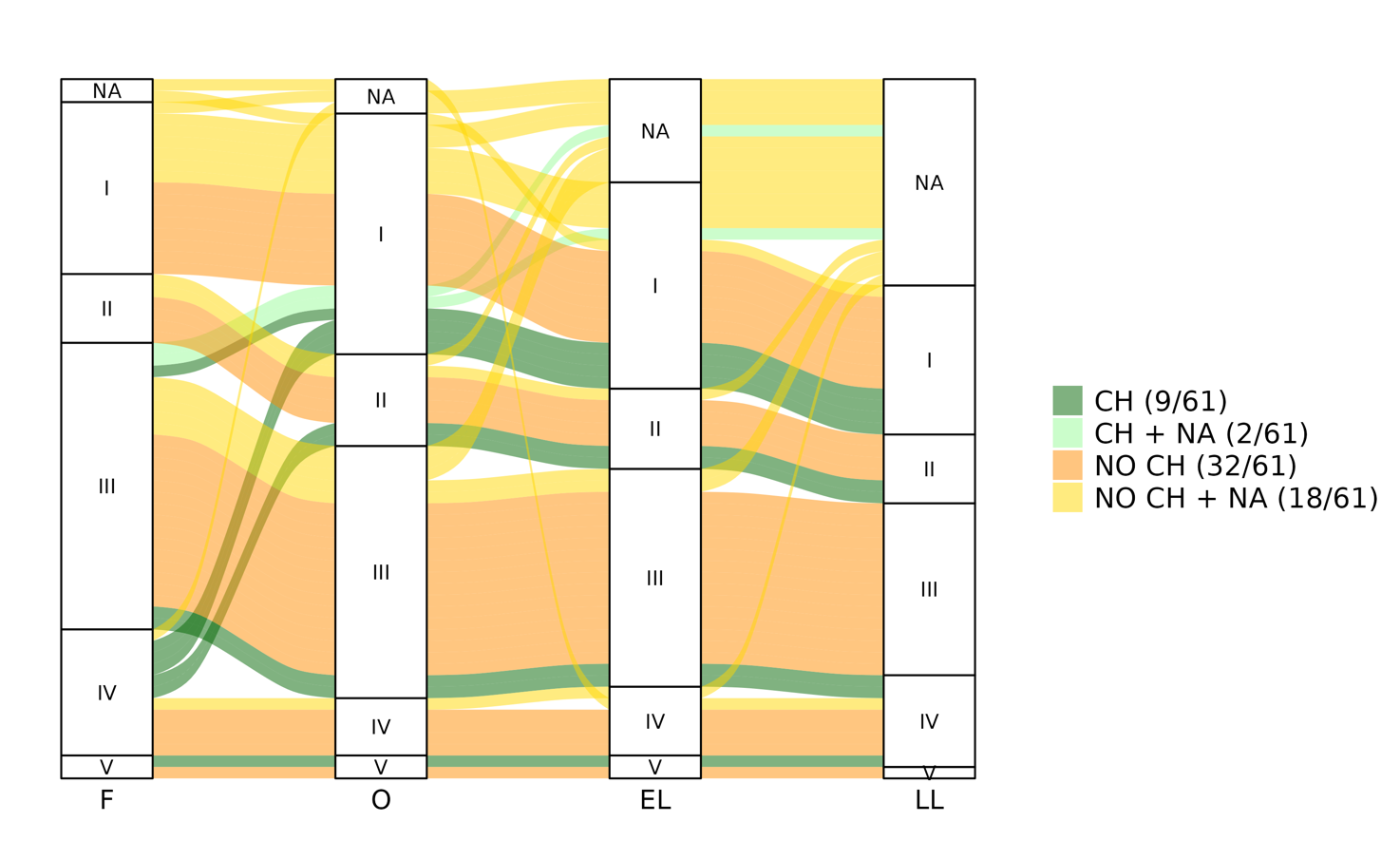


**Figure S17: Correlation of bacteria relative abundances and sex hormones in the four menstrual cycle phases**

These scatterplots visualize the correlation between bacteria relative abundance (CLR-transformed and adjusted for technical covariates) and sex hormones (unadjusted) for each menstrual cycle phase. Plots were generated for all 14 bacteria-sex hormones pairs that resulted significant in model ii) (see **Material and methods**) from **(A)** to **(P)**.

A
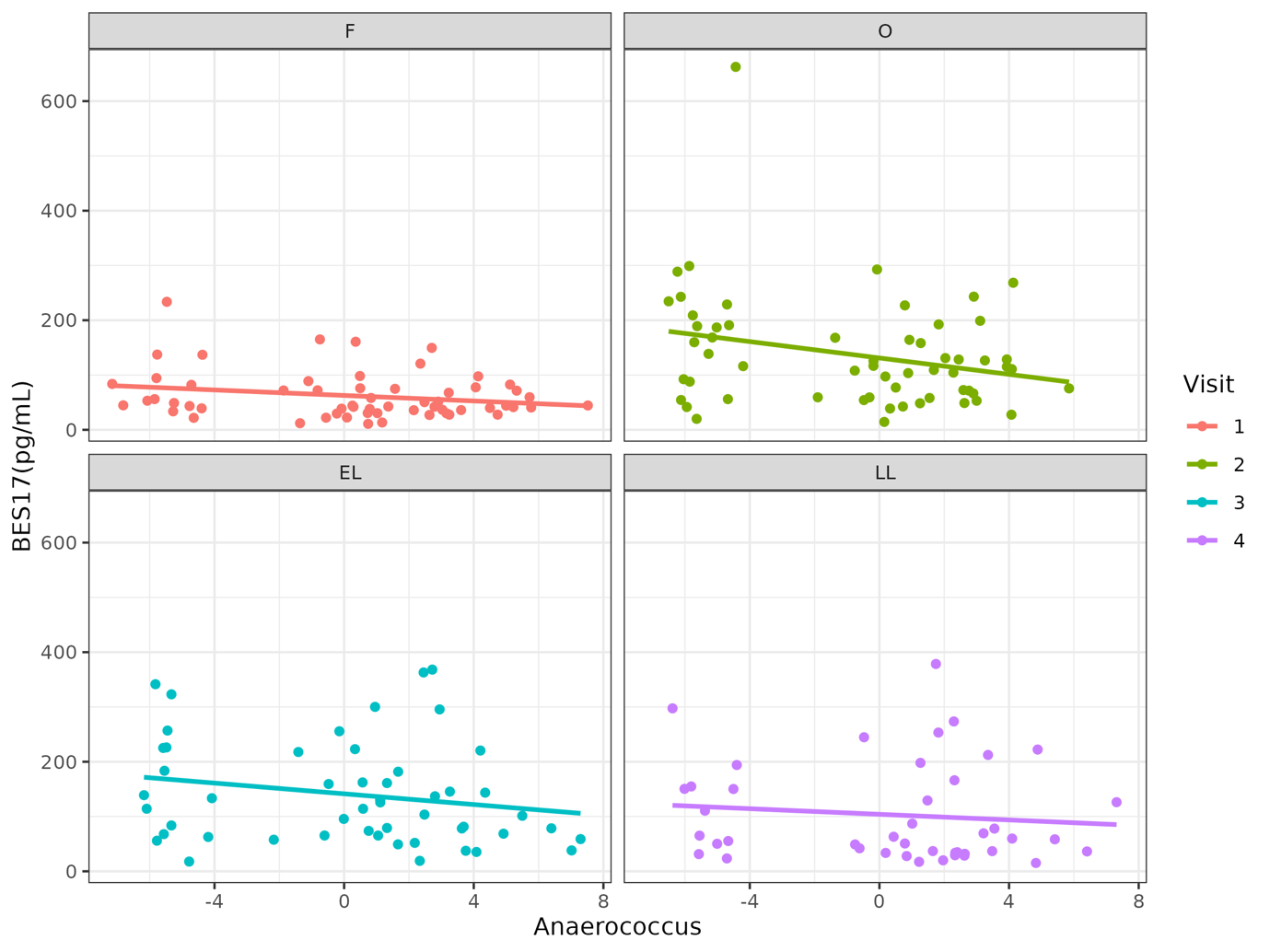


B
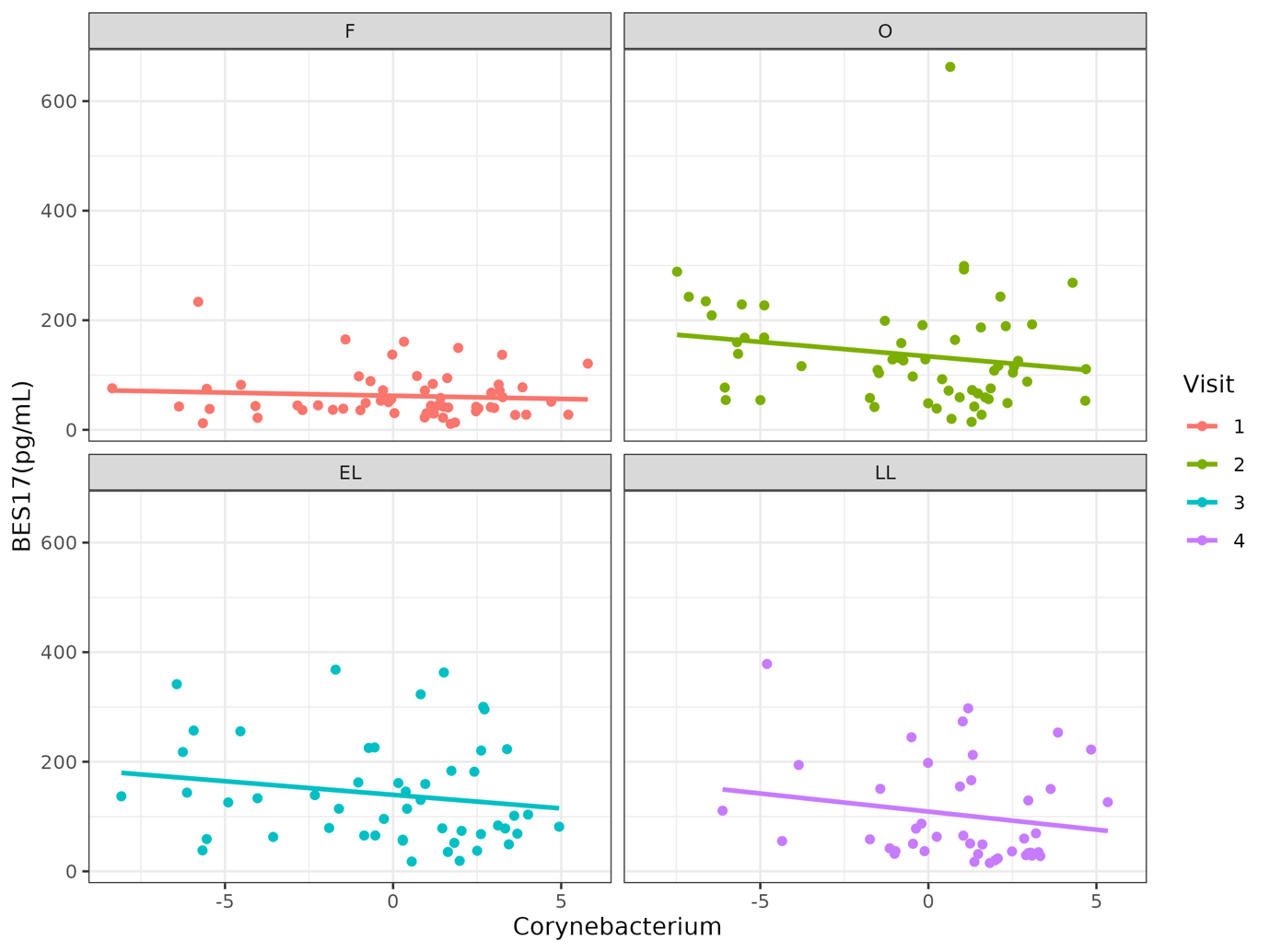


C
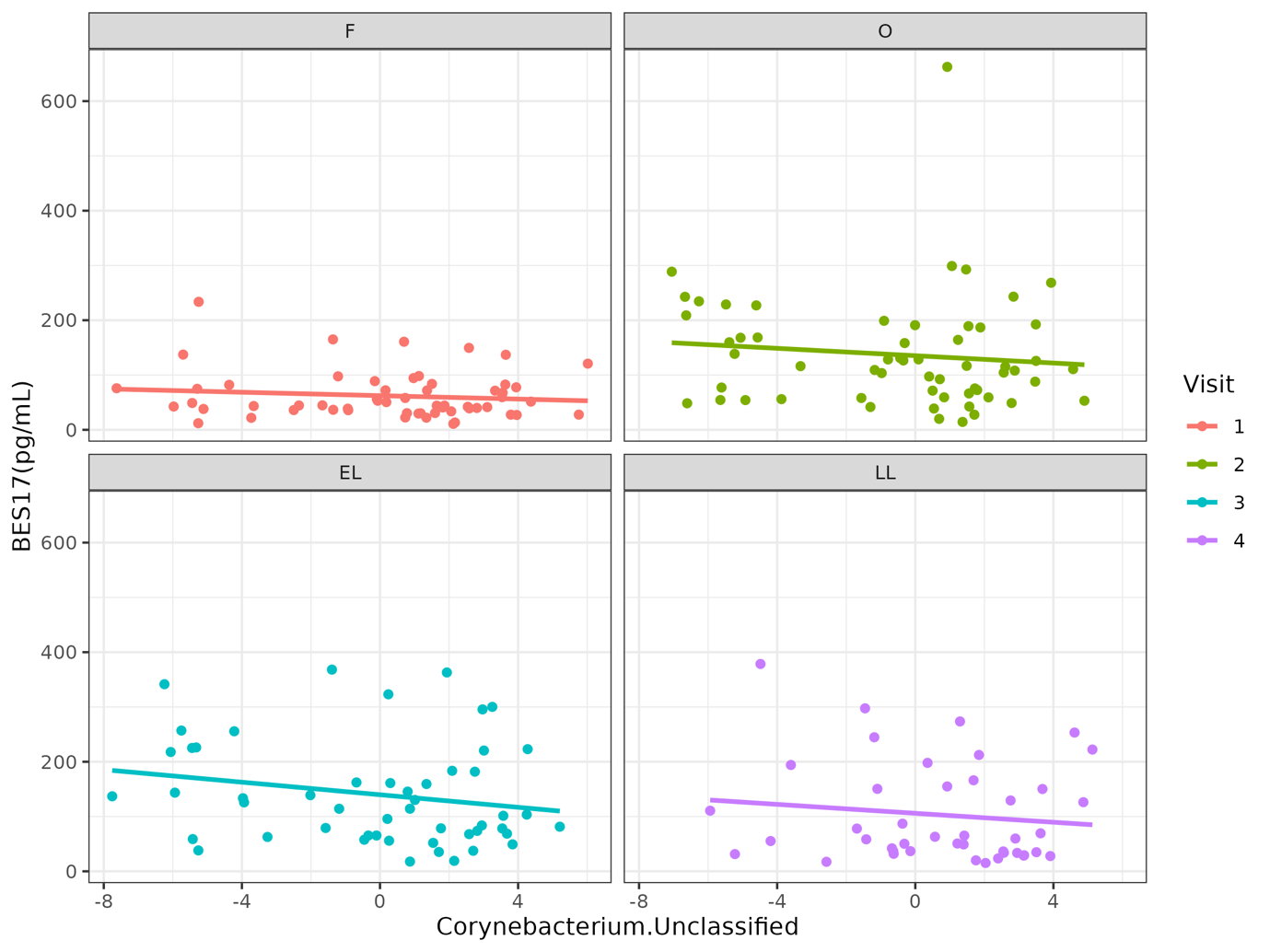


D
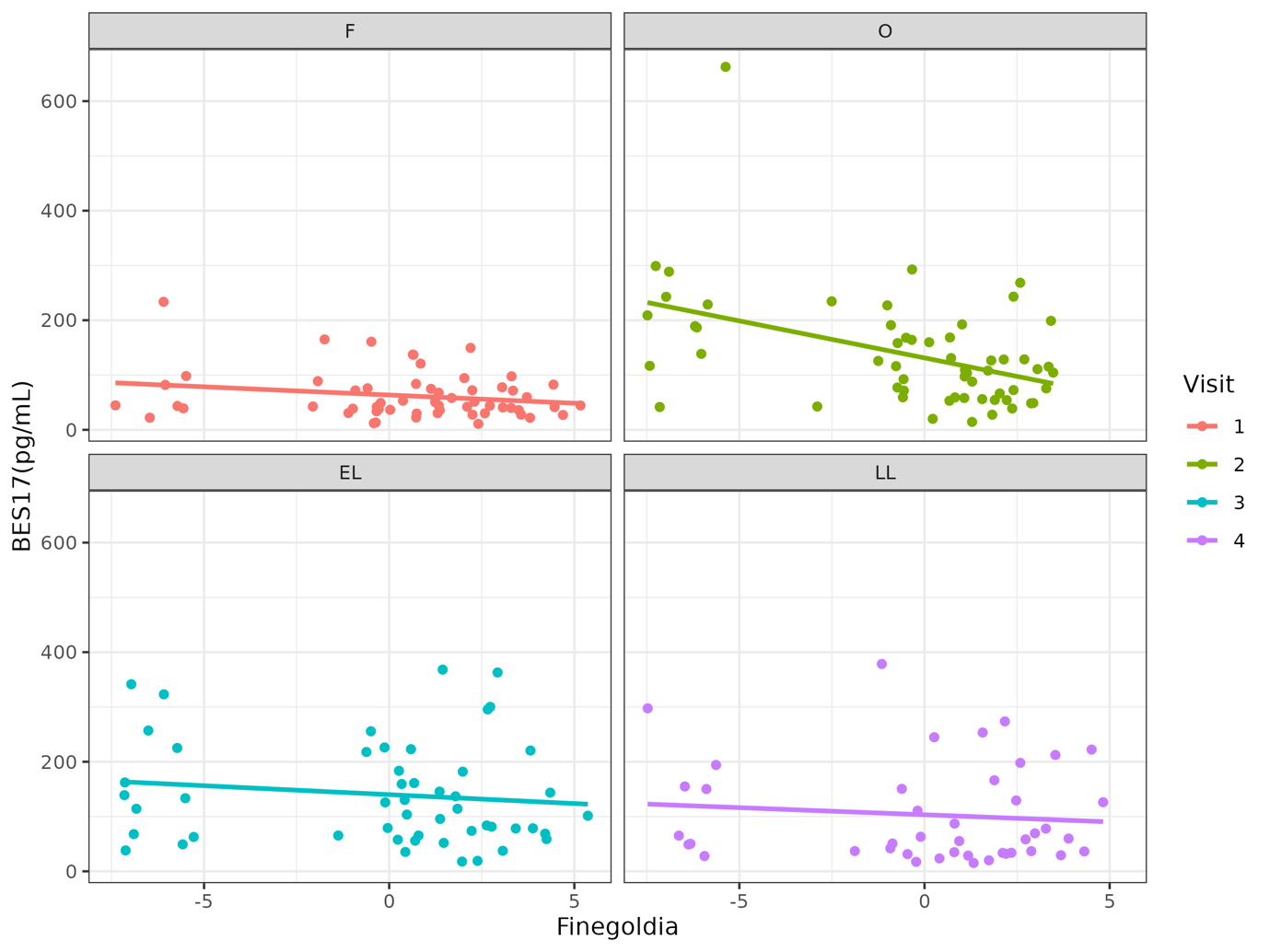


E
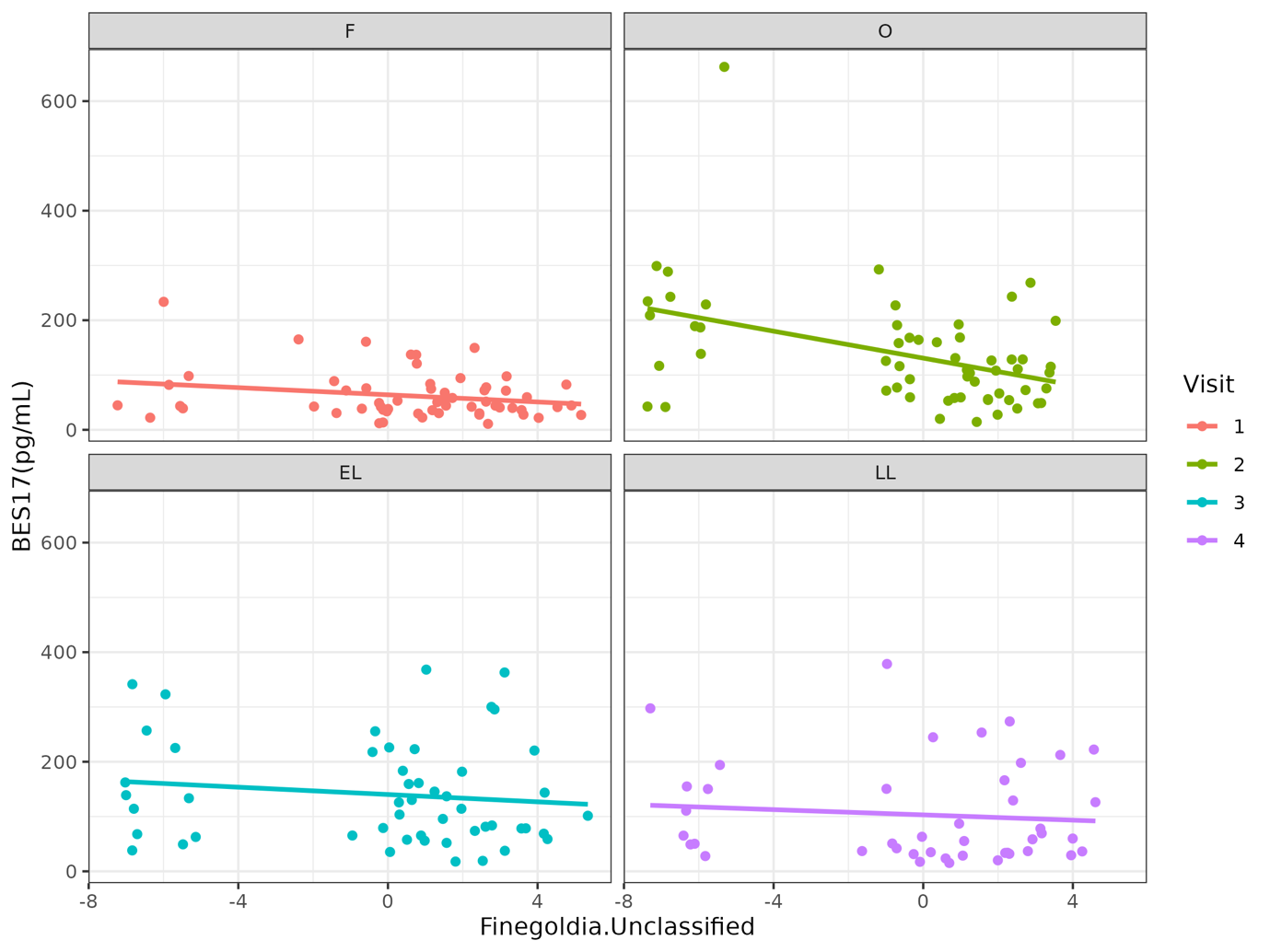


F
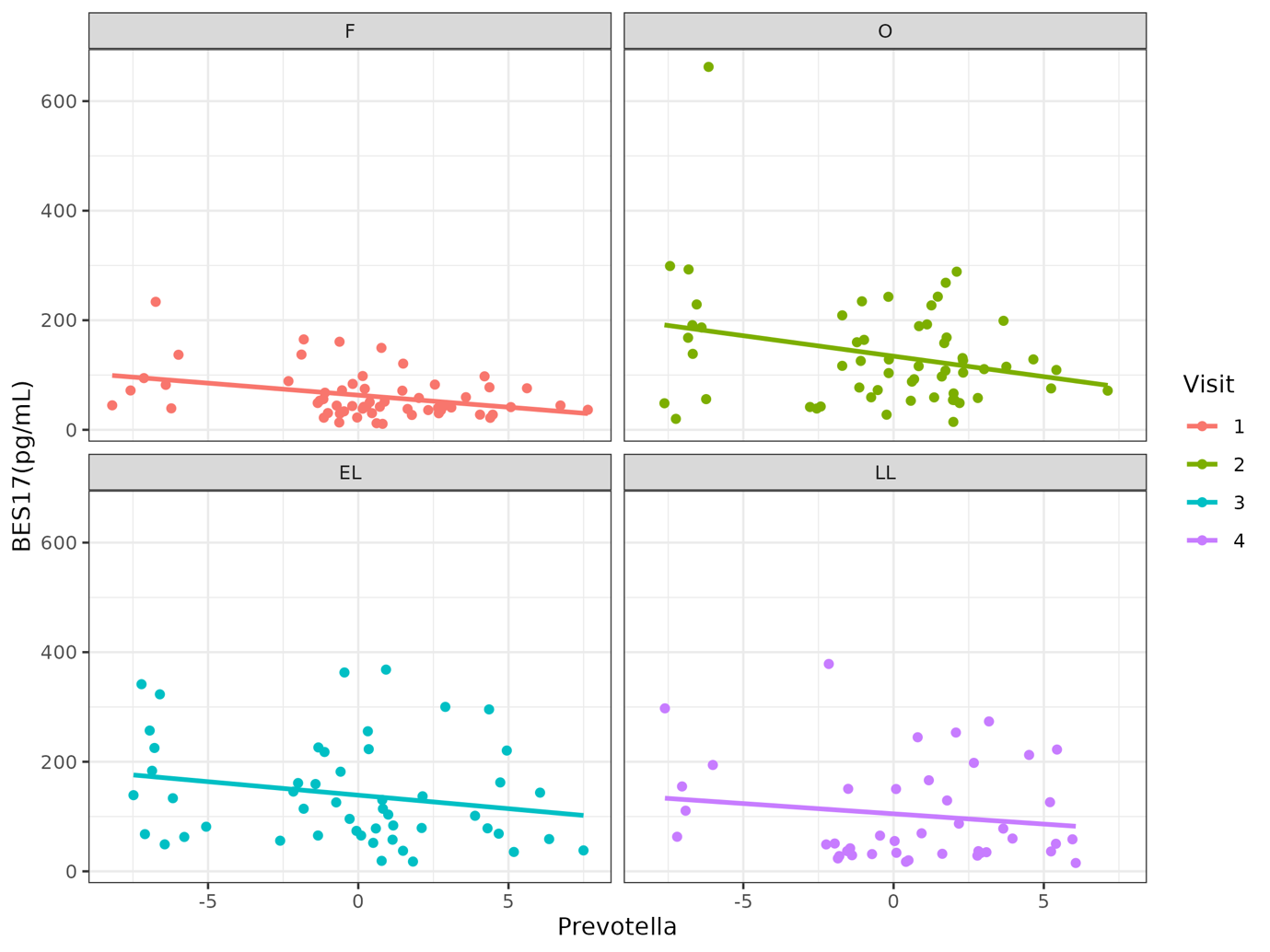


G
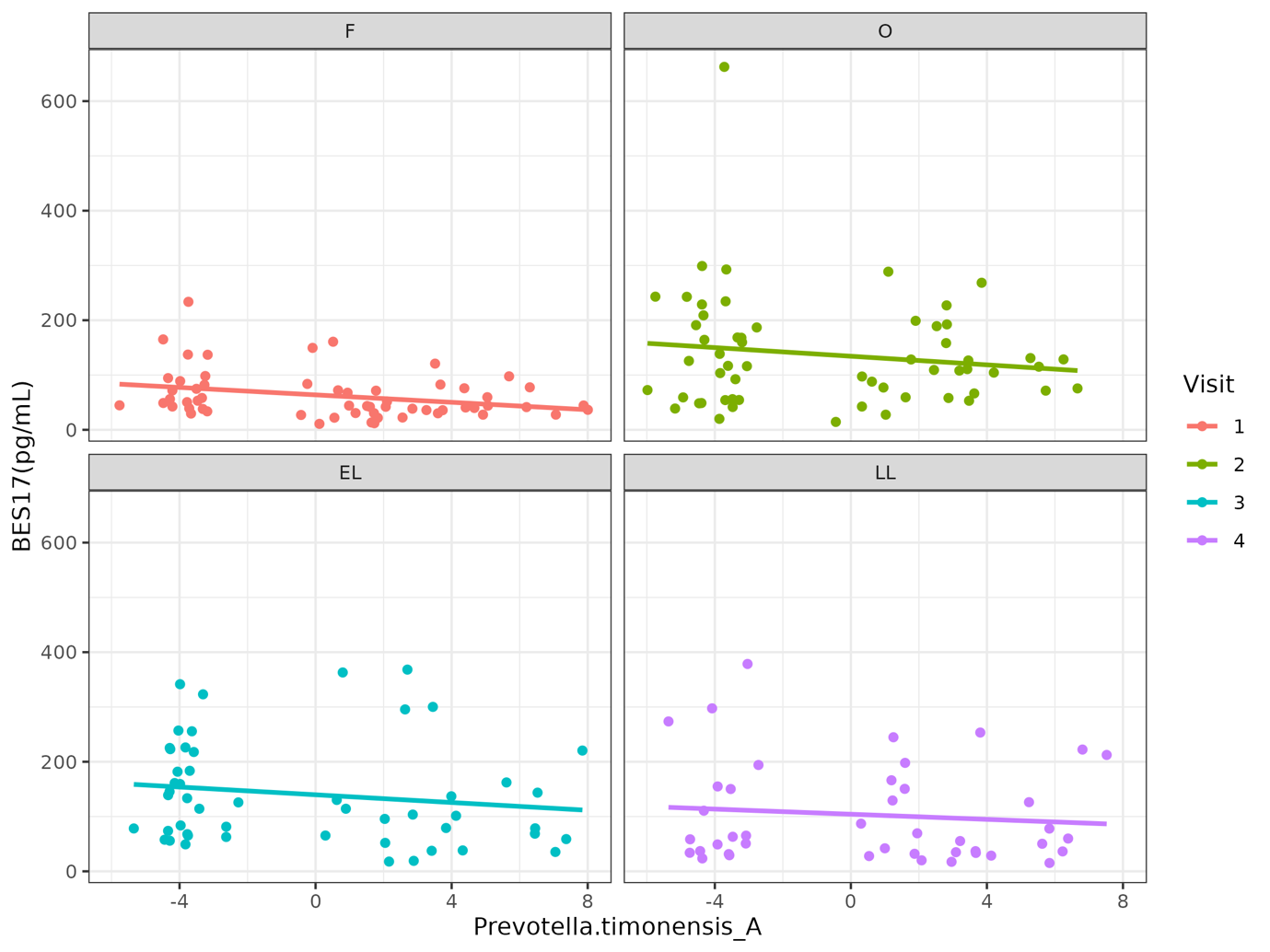


H
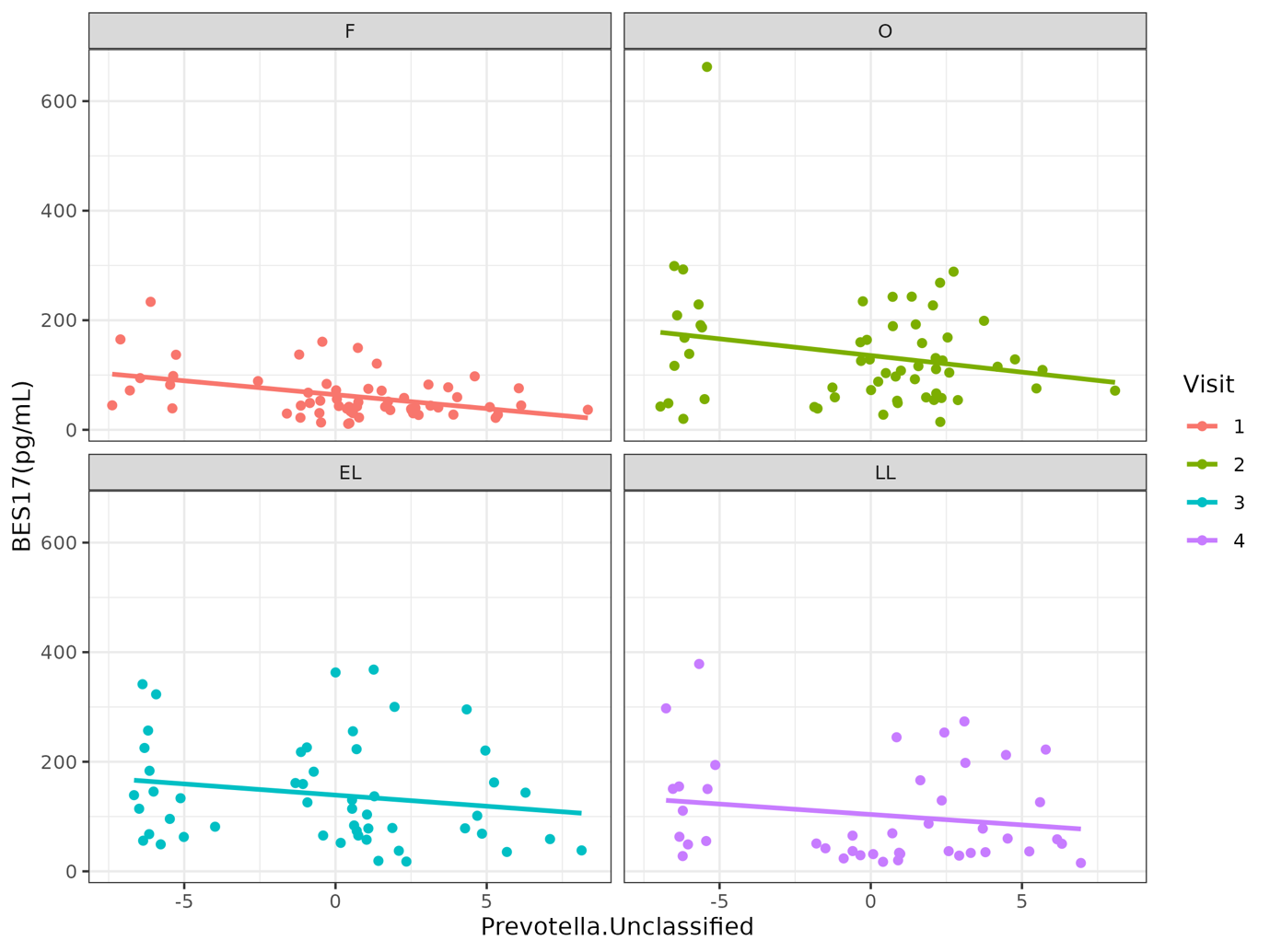


I
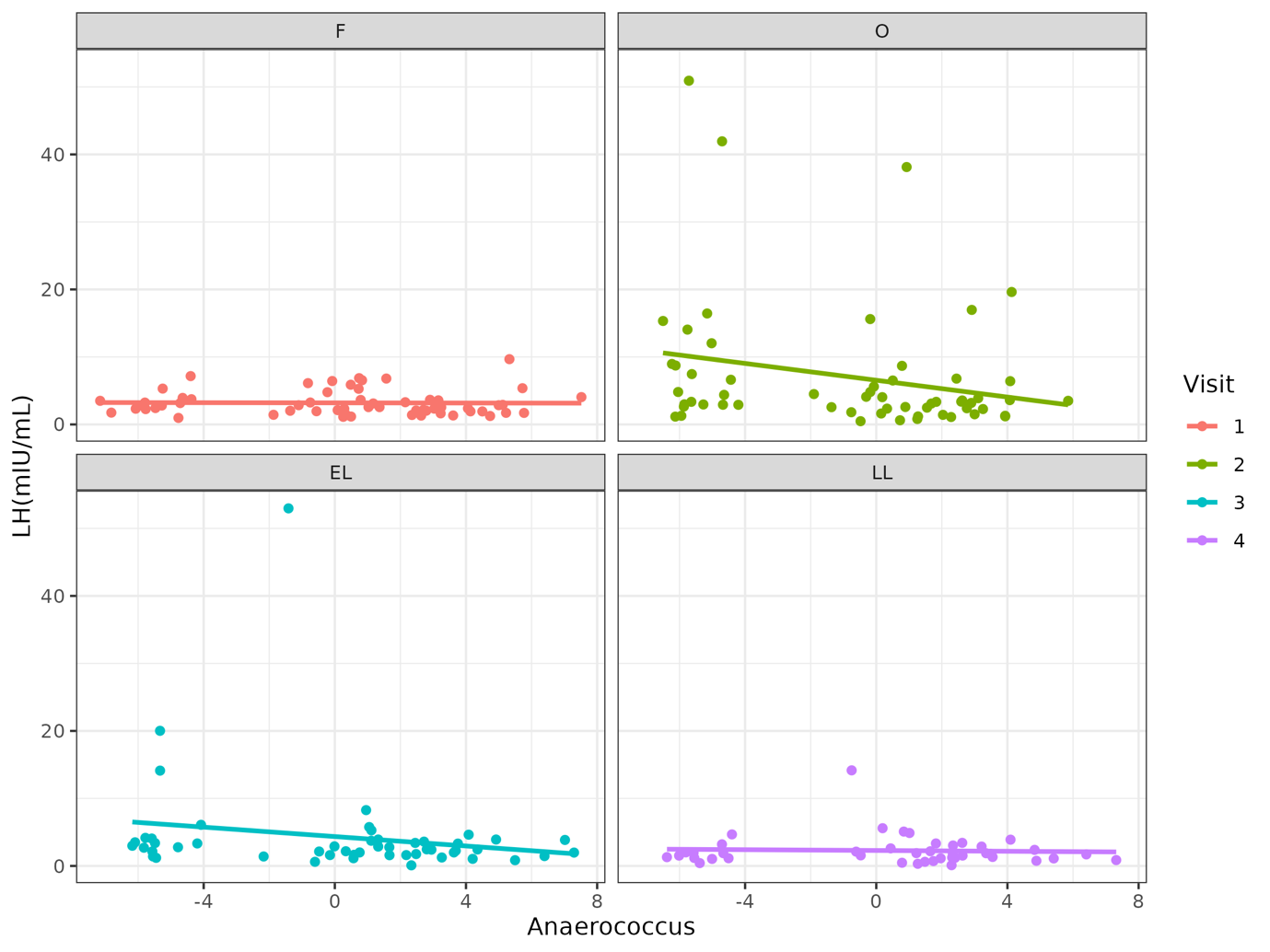


L
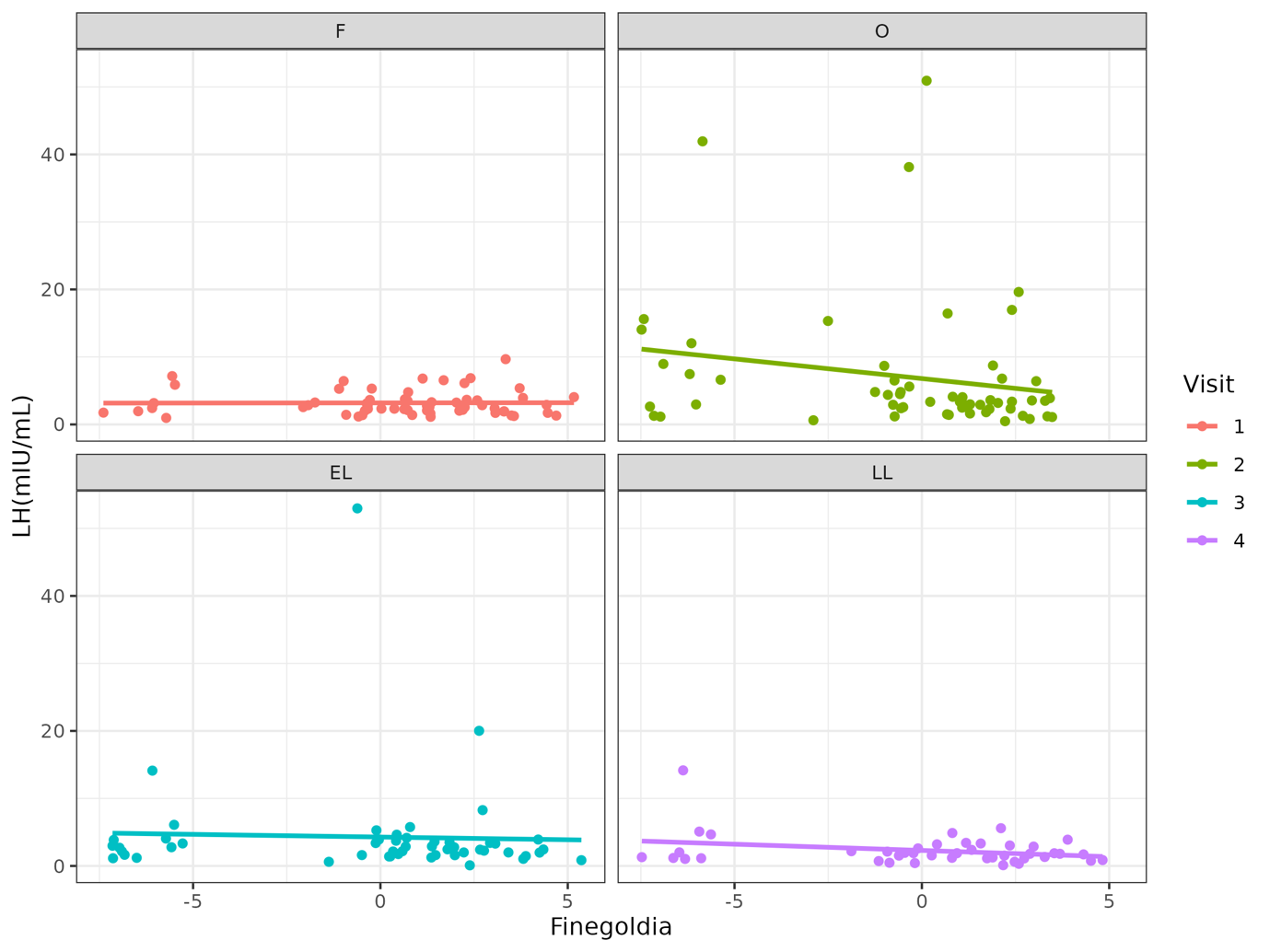


M
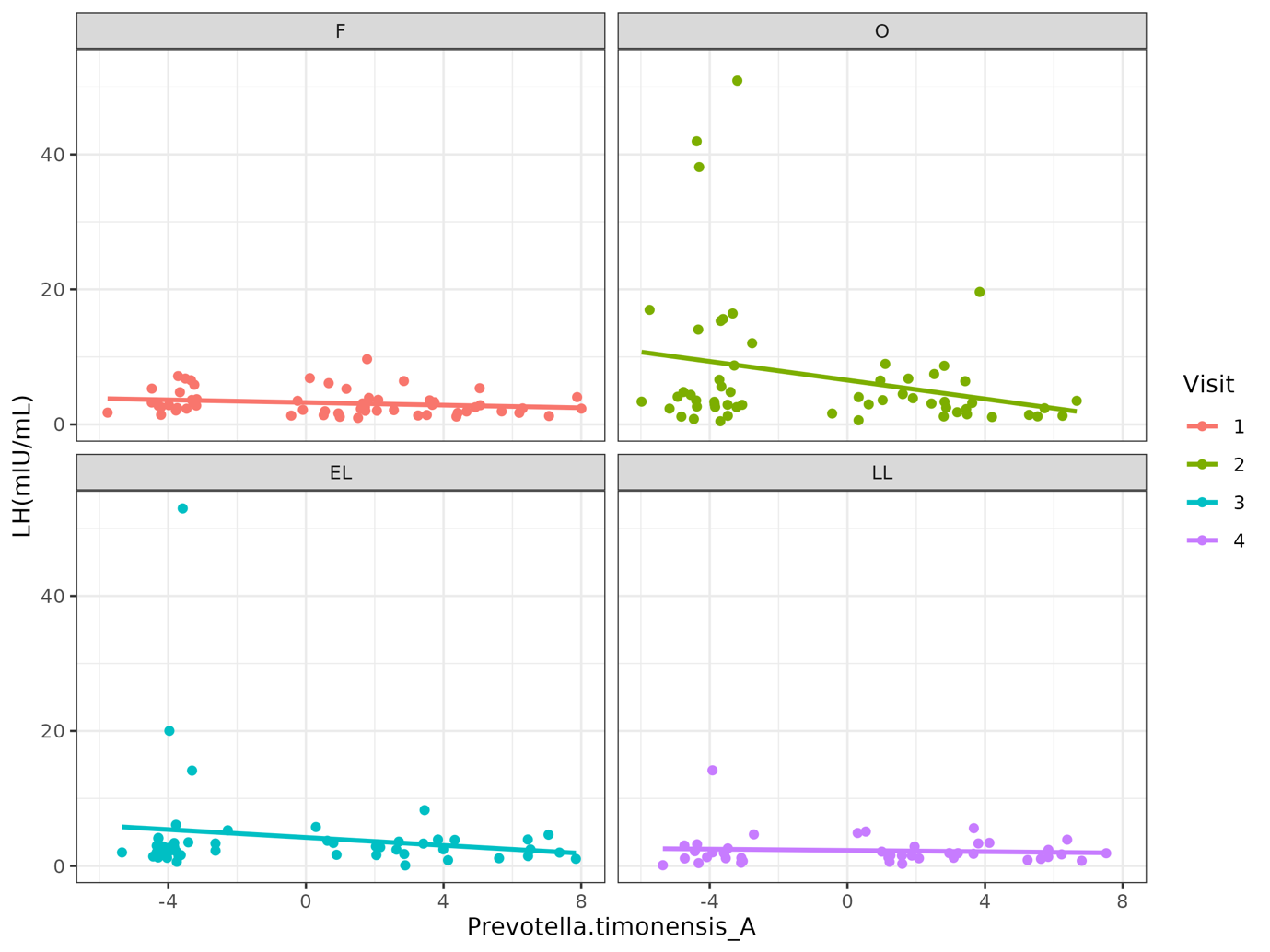
N
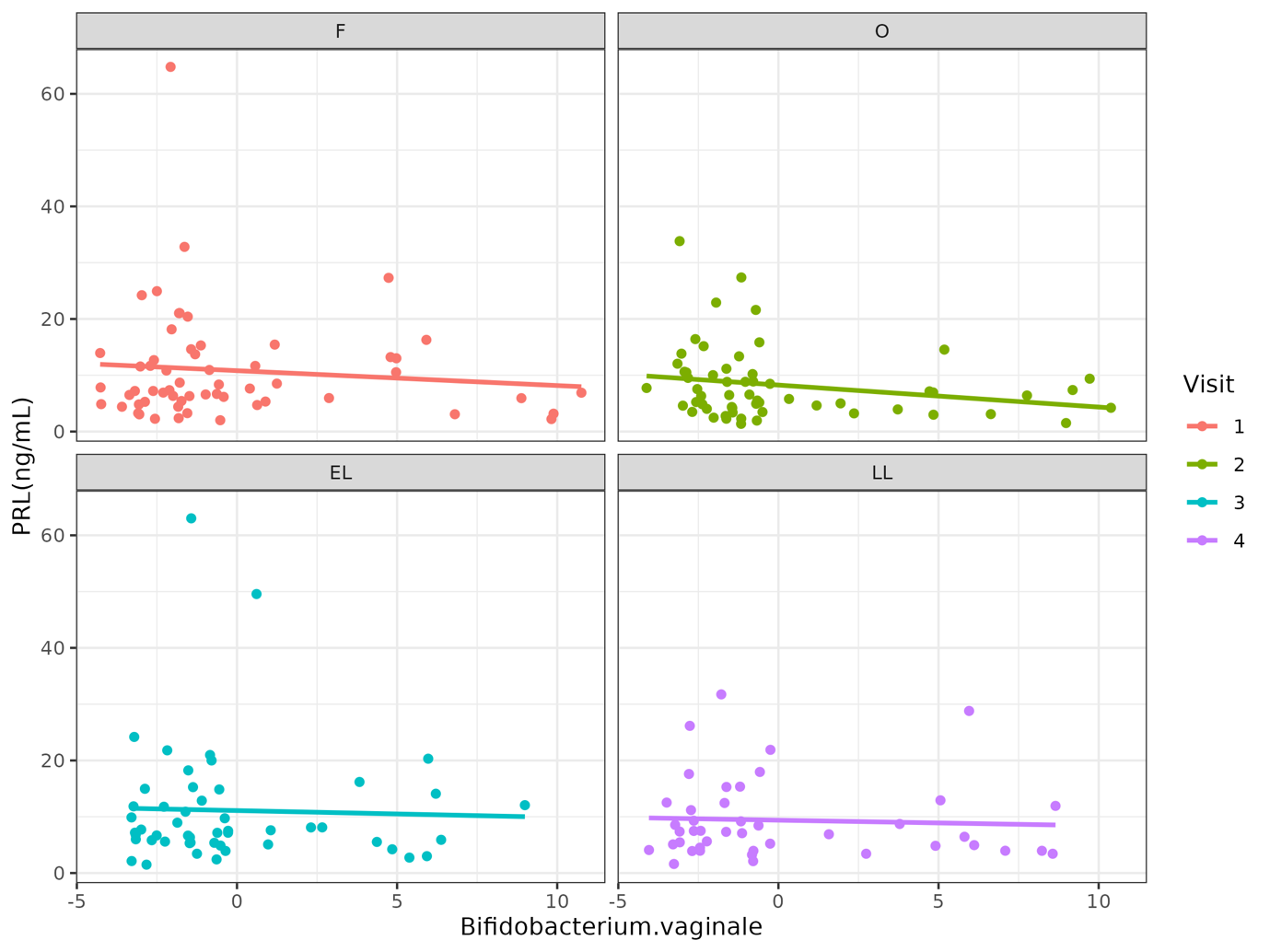


O
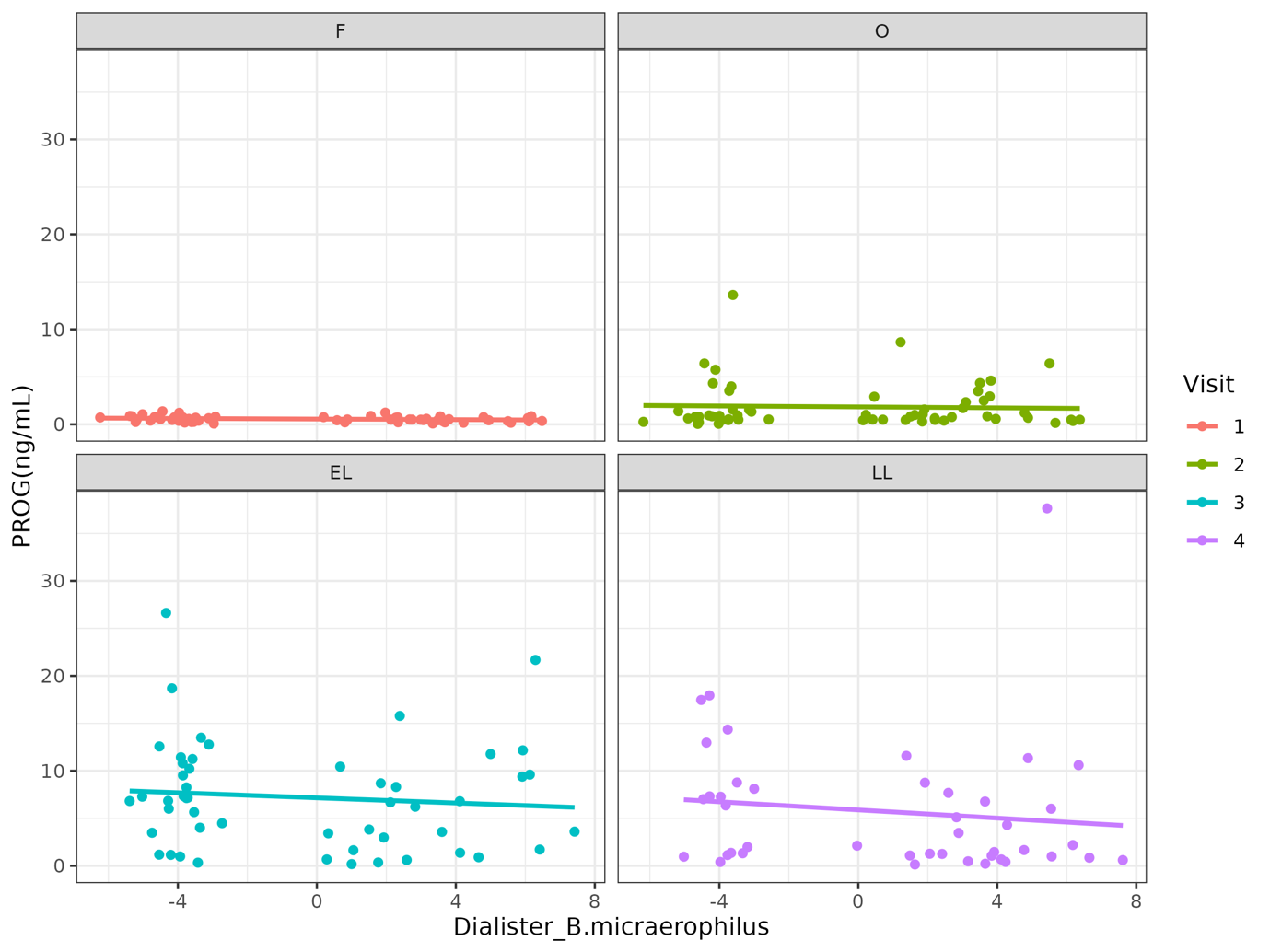


P
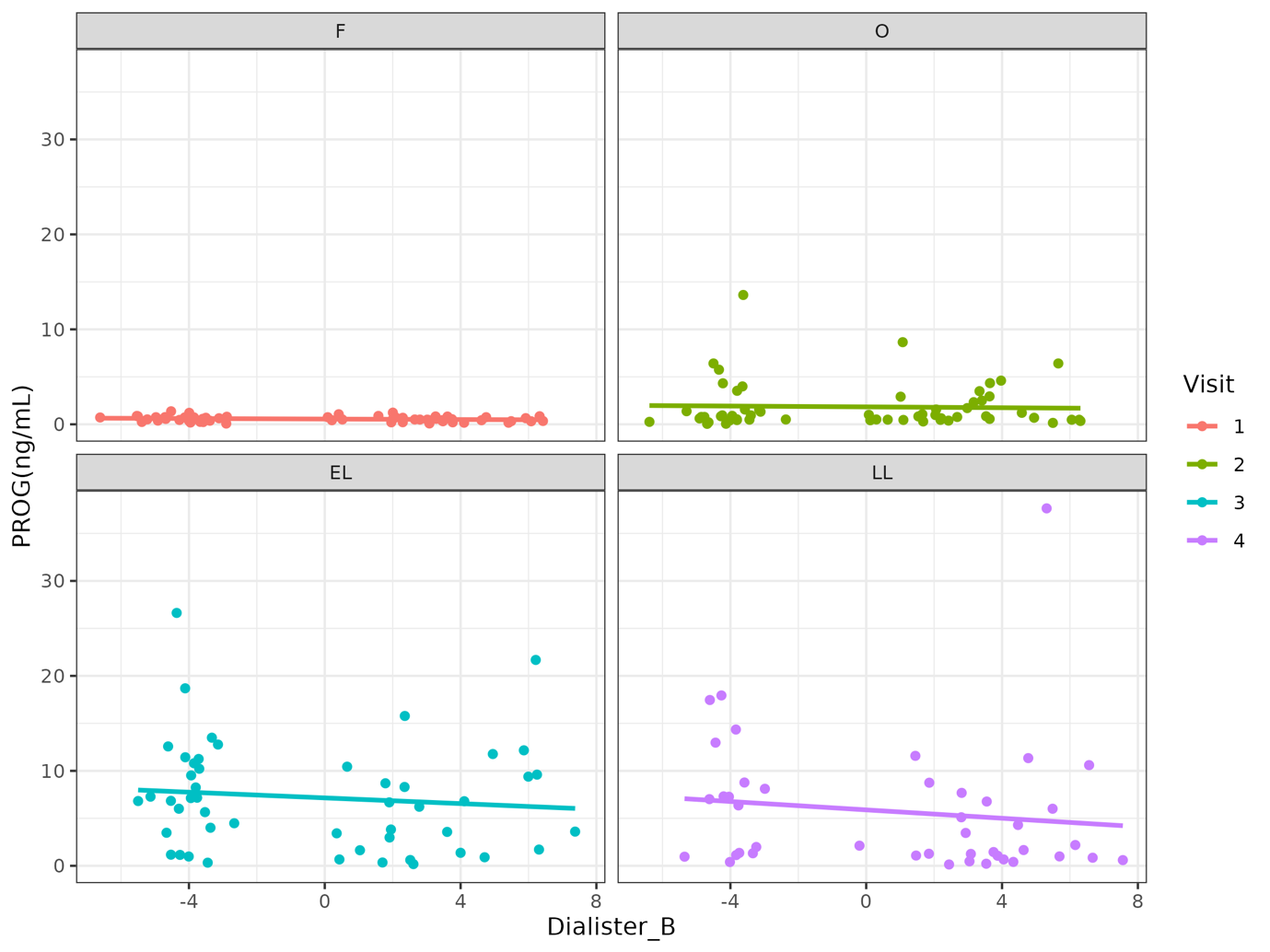

Supplement: Supplemental figures — Figures S1 to S17. [file msystems.00983-25-s0001.docx]
